# Ultraviolet B Exposure Does Not Influence the Expression of YAP mRNA in Human Epidermal Keratinocytes—Preliminary Study

**DOI:** 10.3390/biomedicines13030596

**Published:** 2025-03-01

**Authors:** Igor Aleksander Bednarski, Izabela Dróżdż, Magdalena Ciążyńska, Karolina Wódz, Joanna Narbutt, Aleksandra Lesiak

**Affiliations:** 1 Dermatology, Pediatric Dermatology and Dermatological Oncology Clinic, Medical University of Łódź, 91-347 Łódź, Poland; igor.bednarski@umed.lodz.pl (I.A.B.); joanna.narbutt@umed.lodz.pl (J.N.); 2 Department of Neurology, Medical University of Łódź, 90-419 Łódź, Poland; 3 Department of Clinical Genetics, Medical University of Łódź, 92-213 Łódź, Poland; izabela.drozdz@umed.lodz.pl; 4 Department of Proliferative Diseases, Nicolaus Copernicus Multidisciplinary Centre for Oncology and Traumatology, Medical University of Łódź, 93-513 Łódź, Poland; magdalena.ciazynska@umed.lodz.pl; 5 Laboratory of Molecular Biology, Vet-Lab Brudzew, 62-720 Brudzew, Poland; wodz.karolina@gmail.com

**Keywords:** ultraviolet B, Hippo pathway, YAP, skin carcinogenesis

## Abstract

**Background:** The causal relationship between exposure to ultraviolet radiation and the development of skin cancers requires constant research for possible orchestrating mechanisms. In recent years, the Hippo pathway, along with its effector protein YAP, became implicated in cutaneous carcinogenesis; however, Hippo pathway regulation by ultraviolet radiation has not been described thoroughly. In order to address this issue, we focused on how different doses of ultraviolet B affect Hippo signaling pathway components and its upstream regulators, JNK1/2 and ABL1, in human keratinocytes. Additionally, we decided to determine how silencing of YAP influences Hippo pathway component expression. **Methods:** Primary epidermal keratinocytes were irradiated using UVB lamps with increasing doses of ultraviolet B radiation (including 311 nm UVB). Real-time PCR was used to determine the mRNA levels of each investigated gene. The experiment was then performed after YAP silencing using siRNA transfection. Additionally, we determined the mRNA expression of Hippo pathway components in an A431 cSCC cell line. **Results:** We observed that YAP mRNA expression in the A431 cell line was insignificant in comparison to control, while in the case of LATS1/2, a significant increase was noted. UVB irradiation did not change the levels of YAP mRNA expression in human epidermal keratinocytes. LATS1, LATS2, ABL1 and MAP4K4 mRNA expression was significantly upregulated after UVB irradiation in non-YAP-silenced keratinocytes in a dose-dependent manner, while after YAP silencing, only LATS2 and ABL1 showed significant mRNA upregulation. The 311 nm UVB irradiation resulted in significant, dose-dependent mRNA upregulation in non-YAP-silenced keratinocytes for LATS1, ABL1 and MAP4K4. After YAP silencing, a significant change in mRNA expression was present only in the case of ABL1. **Conclusions:** YAP mRNA expression does not significantly increase after exposure to UVB; however, it upregulates the expression of its proven (LATS1/2, JNK1/2) regulators, suggesting that in real-life settings, UV-induced dysregulation of the Hippo pathway may not be limited to YAP.

## 1. Introduction

Skin, the outermost layer of the human body, provides a protective barrier against harmful environmental factors, including ultraviolet radiation (UVR) [1]. Despite several autoregulatory mechanisms alleviating its destructive properties, an excessive exposition to UVR damages the skin and is essential in the development of non-melanoma skin cancers (NMSCs) and malignant melanoma [2,3,4]. UVR consists of three wavelengths with distinct biological effects. UVA (315–400 nm) is barely absorbed by human DNA and penetrates below the epidermis, causing degeneration of extracellular matrix proteins. UVB (280–315 nm) reaches superficial parts of the epidermis and induces photochemical reactions and mutations within the DNA, while UVC (100–280 nm), although highly mutagenic, generally does not reach Earth’s surface [2,4]. The complex interplay between UVA/UVB, combined with their cumulative radiation dose, genetic susceptibility and activation of different signaling pathways, results in disturbances in skin homeostasis and, eventually, in carcinogenesis [4].

To date, several pathways have been implicated in the process of skin carcinogenesis, e.g., Sonic Hedgehog [5] or TGF-β/Smad [6]; however, in recent years, the Hippo signaling pathway emerged as another possible key player in the development of cutaneous cancers [7,8]. The Hippo pathway, identified in 1995 [9] in overgrown phenotypes of *Drosophila melanogaster*, is an evolutionarily conserved pathway responsible for tissue growth and cellular differentiation [10]. Almost three decades of extensive research also proved its involvement in tumorigenesis by promoting epithelial-to-mesenchymal transition (EMT), apoptosis evasion and malignant transformation [11,12,13]. While being implicated mostly in the pathogenesis of hepatocellular carcinoma, glioblastoma and non-small-cell lung carcinoma [14], recent studies provided multiple proofs that aberrant Hippo signaling contributes to the development of cutaneous tumors and precancerous lesions, including basal [15,16] and squamous cell carcinomas [17,18], malignant melanoma [19], Bowen’s disease [18] and actinic keratosis [20].

Signal transduction in the Hippo pathway relies on the phosphorylation cascade formed by serine/threonine kinases MST1/MST2 (mammalian Ste20-like kinases 1/2) with their co-factor SAV1 (Salvador family WW domain-containing protein 1), LATS1/LATS2 (large tumor suppressor 1/2) and effector protein YAP (Yes-associated protein) [21]. MST1/2 and SAV1 fold a complex that phosphorylates LATS1/2. In the next step, activated LATS1/2 phosphorylates YAP in S127 serine residue, which is followed by an attachment of 14-3-3 protein to YAP and its sequestration in the cytoplasm [12]. Conversely, a lack of YAP phosphorylation or MST/LATS suppression enables YAP relocalization to the cellular nucleus [12]. As YAP lacks a DNA-binding domain, it binds with TEAD (transcriptional enhancer factor domain) transcriptional factors, starting the transcriptional activity of target genes, including *CYR61*, *CTGF*, *MYC* and *AXL* [22], thus augmenting cell proliferation and survival [23]. While this phosphorylation cascade is considered canonical, distinct upstream regulators of the Hippo pathway may switch its signaling and promote apoptosis. Emerging evidence highlighted that JNK1/2, ABL1 or MAP4K4 serve as potent modulators of Hippo signaling under genotoxic stress; however, reciprocal crosstalk between them and the Hippo pathway remains elusive.

The Jun N-terminal kinases (JNKs) belong to the mitogen-activated protein kinase (MAPK) family, which are activated in response to a vast array of stimuli, i.e., inflammatory cytokines, growth factors, disturbances in cell polarity and ultraviolet radiation and, alike the Hippo pathway, JNK signaling alternates between pro-proliferative and proapoptotic state depending on the context [24]. Additionally, upon exposure to UVC, JNK phosphorylates YAP in S317 and T362 residues and triggers apoptosis [25]. MAP4K4 is serine/threonine kinase-belonging, similar to MST1/2 and Ste20-like protein kinases. It has been shown that MAP4K4, in the absence of MST1/2, phosphorylates LATS1/2, thus enabling YAP sequestration in the cytoplasm [26]. Moreover, growing evidence shows MAP4K4 upregulation, e.g., in pancreatic ductal adenocarcinoma [27], hepatocellular carcinoma [28] or lung adenocarcinoma [29], but the role of MAP4K4 in skin cancer development is to be elucidated. Lastly, downstream Hippo signaling could be regulated by tyrosine kinase ABL1, which, in a physiological state, controls cell growth and survival, but upon DNA damage, ABL1 phosphorylates YAP at Tyr357 [30]. Tyrosine-phosphorylated YAP binds with p73 and induces proapoptotic gene expression, therefore abolishing YAP proliferative activity [31].

The causal relationship between exposure to UVR and the development of NMSC, as well as the role of YAP in cancer initiation and progression, implies that UVB could activate YAP or suppress Hippo pathway signaling. In order to address this issue, we focused on how different doses of UVB affect Hippo signaling pathway components and its upstream regulators, JNK1/2 and ABL1, in human keratinocytes. Since there is almost no information about the potential involvement of MAP4K4 in UV-induced skin carcinogenesis, we also evaluated its mRNA expression under the influence of UVB. Moreover, we decided to determine how silencing YAP influences Hippo pathway component expression under the same study conditions. Lastly, due to the high efficacy of narrowband UVB in the therapy of various skin disorders, especially psoriasis, we performed experiments again using irradiation with narrowband UVB.

## 2. Materials and Methods

### 2.1. Cell Cultures

We used commercially available human keratinocytes and cutaneous squamous cell carcinoma cell lines. Primary epidermal keratinocytes (PCS-200-011, ATCC, Manassas, VA, USA) were grown in Dermal Cell Basal Medium supplemented with keratinocyte growth components (Bovine Pituitary Extract—BPE, rh TGF-α, L-glutamine, hydrocortisone hemisuccinate, insulin, epinephrine, apo-transferrin). Cell line A431 (CRL-1555, ATCC, Manassas, VA, USA) was grown in EMEM (EBSS) supplemented with 2 mM glutamine, 1% non-essential amino acids (NEAAs) and 10% fetal bovine serum (FBS). TE 354.T (CRL-7762, ATCC, Manassas, VA, USA) cells were grown in Dulbecco’s Modified Eagle’s Medium with 10% fetal bovine serum (FBS). All cell lines were cultured at 37 °C in a 5% CO_2_ incubator. Third passage cells were used for the irradiation and siRNA transfection.

### 2.2. UVB and UVB 311 nm Light Exposure

Keratinocytes were irradiated using TL20W/01 narrowband UVB lamps with spectral output of 305–315 nm (Philips, Hamburg, Germany) and TL20W/12 UVB lamps with spectral output of 285–350 nm (Philips, Hamburg, Germany). The exact dose and irradiation times were calculated according to the manufacturer’s protocols. For UVB treatments, cells on 24-well plates of density 6 × 10^4^ cells per well were washed twice with sterile PBS and irradiated with:

- 0.005, 0.01, 0.02, 0.04 J/cm^2^ of UVB;
- 0.01, 0.02, 0.05, 0.1 J/cm^2^ of 311 nm UVB.

No PBS was added to cells for UVB irradiation (short periods for UVB radiation). All irradiation cells were kept cold on ice to avoid excessive warming of cells. After UV irradiation, PBS was removed, and all cells were incubated in culture medium for 48 h.

### 2.3. siRNA Transfection

Before siRNA transfection, keratinocytes were seeded at 6 × 10^4^ cells/well in 24-well plates. After 24 h incubation at 37 °C, the cells were transfected with siRNA against YAP (Qiagen, Valencia, CA, USA). All transfection reagents, including siRNA, negative control, positive control and Transfection Reagent (Qiagen, Valencia, CA, USA), were used according to the manufacturer’s instructions. Cells were incubated in medium overnight. After siRNA interfering, keratinocytes were irradiated with UVB and 311 nm UVB under the same conditions as cells without siRNA transfection.

### 2.4. Quantitative Real-Time-PCR

Real-time PCR was used to determine the mRNA levels of each investigated gene. Total RNA from non-irradiated cells (constitutive mRNA expression), irradiated cells, siRNA transfection, and irradiated cells with UVB and UVB-311 nm were isolated using an AllPrep DNA/RNA/Protein Mini Kit (Qiagen, Valencia, CA, USA) according to the manufacturer’s protocol. RNA was reverse transcribed using a High-Capacity cDNA Reverse Transcription Kit (ThermoFisher, Waltham, MA, USA) according to the manufacturer’s protocol.

The expression of all mRNAs was performed using TaqMan^®^ probes dyed FAM (Table 1). Gene-specific PCR products were measured continuously using the LightCycler480 System (Roche, Indianapolis, IN, USA) for 40 cycles. The amplification conditions were as follows: polymerase activation at 95 °C for 10 min, followed by 40 cycles of amplification: 95 °C for 15 s and 60 °C for 60 s. Target gene expression was normalized between different samples based on the values of the endogenous control expression for each cDNA sample. Quantitative analysis of data was performed according to the ∆∆CT method. The RT-qPCR amplification of each gene of interest (GOI) was compared to that of GADPH, a house-keeping reference gene (HKG), and ΔCt values were determined (ΔCt = GOICt − HKGCt). The results were analyzed according to the 2^−ΔΔCt^ method. The results were analyzed using GraphPad Prism 10.0 software (GraphPad Software, La Jolla, CA, USA). Intergroup comparisons were performed with Student’s *t*-tests and Mann–Whitney U tests (for comparisons between two groups) or appropriate ANOVA tests (for comparisons of ≥2 groups). Dunnett’s and Dunn’s post-hoc tests were used to compare individual results with the control group. Unless otherwise stated, results are presented as fold change (FC) with geometric standard deviation (fold change, FC, 2^−ΔΔCt^ used interchangeably). Asterisks above the bars indicate the statistical significance level of the post-hoc tests (* *p* < 0.05, ** *p* < 0.001, *** *p* < 0.0001). In all analyses, a significance level of *p* < 0.05 was deemed statistically significant.

## 3. Results

### 3.1. Hippo Pathway and Its Regulator mRNA Expression in A431 Cell Line

**Figure 1:**
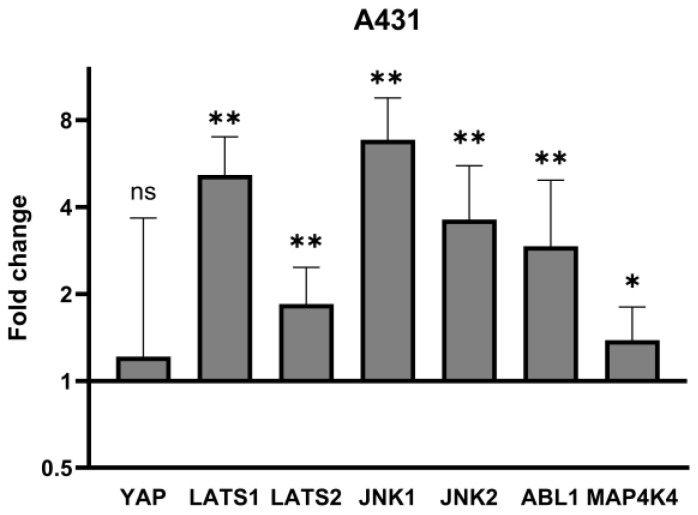
mRNA expression of YAP and its regulators in A431 cell line. Data presented as fold change (columns) with geometric standard deviations (bars). Asterisks above columns indicate level of statistical significance (ns—not significant, * *p* < 0.05, ** *p* < 0.001) in comparison to controls.

In the first step, we measured YAP, LATS1/2, JNK1/2, ABL1 and MAP4K4 gene expression in the A431 cell line to identify which disturbances in the Hippo pathway and their regulators are present in the standardized squamous cell carcinoma cell line. We observed that YAP mRNA expression was insignificant in comparison to control, while in the case of LATS1/2, a significant (5-fold, 1.8-fold, respectively) increase was noted. JNK1, JNK2, ABL1 and MAP4K4 showed significant increases compared to controls (6.8-fold, 3.6-fold, 2.9-fold, 1.4-fold, respectively). Detailed results are presented in Table 2 and Figure 1.

### 3.2. Hippo Pathway and Its Regulator mRNA Expression in Human Epidermal Keratinocytes After UVB Irradiation

**Figure 2:**
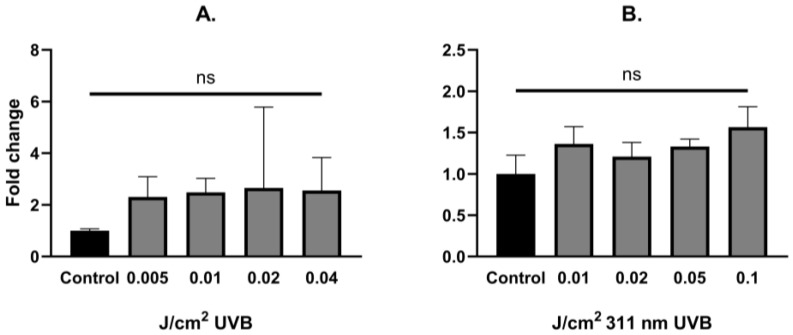
YAP fold change alterations in primary epidermal keratinocytes after exposure to different doses of UVB (**A**) and 311 nm UVB (**B**). Data presented as fold change (columns) with geometric standard deviations (bars) (ns—not significant).

UVB irradiation did not change the levels of YAP mRNA expression in human epidermal keratinocytes (Figure 2A,B). LATS1, LATS2, ABL1 and MAP4K4 mRNA expression was significantly upregulated after UVB irradiation in non-YAP-silenced keratinocytes in a dose-dependent manner (*p* = 0.033, *p* = 0.016, *p* = 0.039, *p* = 0.027, respectively). After YAP silencing, only LATS2 and ABL1 showed significant mRNA upregulation (*p* = 0.042, *p* = 0.003, respectively). Regardless of YAP silencing, no changes in JNK1 and JNK2 mRNA expression were noted after irradiation.

**Figure 3:**
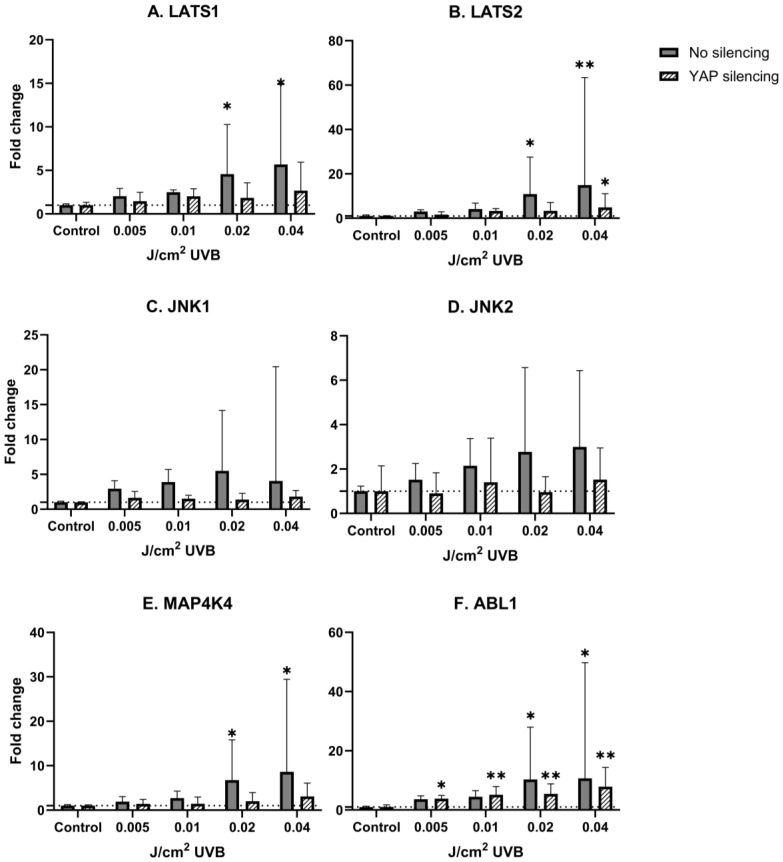
mRNA expression of studied Hippo pathway regulators: LATS1 (**A**), LATS2 (**B**), JNK1 (**C**), JNK2 (**D**), MAP4K4 (**E**), ABL1 (**F**) in primary epidermal keratinocytes (3 repetitions) with preserved (dark grey) and silenced (tilted) expression of YAP under the influence of increasing doses of UVB. Data presented as fold change (columns) with geometric standard deviations (bars). Asterisks above columns indicate level of statistical significance (ns—not significant, * *p* < 0.05, ** *p* < 0.001) in comparison to controls.

**Figure 4:**
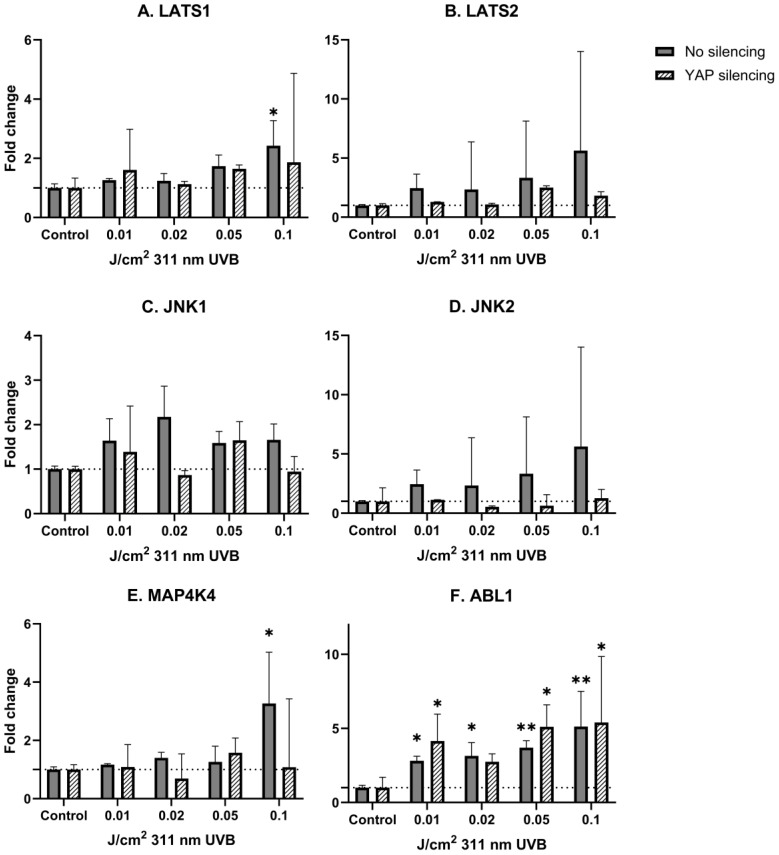
mRNA expression of studied Hippo pathway regulators: LATS1 (**A**), LATS2 (**B**), JNK1 (**C**), JNK2 (**D**), MAP4K4 (**E**), ABL1 (**F**) in primary epidermal keratinocytes (2 repetitions) with preserved (dark grey) and silenced (tilted) expression of YAP under the influence of increasing doses of narrowband UVB. Data presented as fold change (columns) with geometric standard deviations (bars). Asterisks above columns indicate level of statistical significance (ns—not significant, * *p* < 0.05, ** *p* < 0.001) in comparison to controls.

Considering narrowband UVB irradiations, a significant, dose-dependent mRNA upregulation was observed in non-YAP-silenced keratinocytes for LATS1, ABL1 and MAP4K4 (*p* = 0.031, *p* = 0.006, *p* = 0.033, respectively). After YAP silencing, a significant change in mRNA expression was present only in the case of ABL1 (*p* = 0.022). Detailed results are shown in Table 3 and Table 4 and Figure 3 and Figure 4.

## 4. Discussion

Under physiological conditions, the Hippo pathway, along with its downstream effector YAP, has a profound effect on skin development and wound healing by controlling epidermal proliferative capacities, while disruption within its signaling cascade results in excessive cell proliferation and aberrant differentiation, suggesting that Hippo pathway may be involved in the development of keratinocyte-derived tumors, especially cutaneous squamous cell carcinoma (cSCC) [17,32,33,34]. Indeed, YAP overexpression augments cSCC growth and formation, as well as its metastatic capabilities and resistance against treatment [16,35], which has been proven in a number of studies. Schlegelmilch et al. [17], using a murine model, showed that grafting of transgenic skin tissue with constitutional YAP activation on nude mice recipients results in the development of invasive SCC-like tumors with spindle-cell carcinoma properties [17]. Later, the involvement of YAP in the progression from SCC into spindle-cell carcinoma was confirmed by Vincent-Mistiaen et al. [36], who reported that YAP expression is necessary for epithelial-to-mesenchymal transition (EMT), which enhances the invasive ability of cSCC, eventually allowing metastasis [37]. Jia et al. [20], using immunohistochemistry on skin samples taken from different SCC stages, demonstrated that YAP protein expression correlates positively with the cancer stage and found that cytoplasmic and nuclear YAP protein expression was significantly higher in cSCC samples compared to actinic keratosis, Bowen’s disease and healthy skin [20]. In line with these findings, Debaugnies et al. [16] showed that strong nuclear YAP staining is associated with cSCC invasiveness, both in murine and human models. Additionally, analysis of oncogenic YAP effects in SCC cell lines, A431 and SCL-1, revealed that knockdown of YAP resulted in diminished cell growth in both cell lines, while YAP overexpression protected cancer cells from apoptosis induced by 5-fluorouracil [20]. In our study, we have not observed increased YAP mRNA expression in the A431 cell line, whereas levels of LATS1 and JNK1 were significantly upregulated (5.2 and 6.8-fold, respectively), suggesting that in differentiated cSCC (A431), there may be a negative feedback loop suppressing YAP expression or other mechanisms maintaining cSCC malignant properties.

These reports, taken together, imply that cSCC development is somewhat of a YAP-dependent phenomenon. Taking into account that UVR is considered the main environmental risk factor in the development of cSCC, and there is a growing amount of evidence that Hippo signaling could be one of the core regulators in this process, it remains uncertain how UVR influences the Hippo pathway signaling cascade. To date, there is a limited amount of information that YAP expression is explicitly regulated by UVR; nevertheless, some indirect proofs suggest that UV-induced carcinogenesis may be, at least partially, YAP-driven. In a molecular context, the role of YAP could be epitomized as a double-edged blade. While being implicated as a potent oncogene in a variety of cancers by triggering proliferation and apoptosis evasion, YAP could also act as a tumor suppressor by stimulating apoptosis. This dual role of YAP depends on its phosphorylation and cellular location in which JNK1/2 kinases may play a role. It is established that JNK1/2 kinases are activated in response to genotoxic stress, including UVR [38,39]; however, the relationship between JNK and the Hippo pathway is not fully described yet. In the study performed by Lee and Yonehara [40], both irradiation with UV, as well as exposure to cisplatin resulted in multisite phosphorylation of YAP induced by p38/JNK, which protected the cells against apoptosis [40]. Tomlinson et al. [25] showed that exposure to UVC results in robust phosphorylation of YAP by JNK1/2 in S317 and T362 residues, which protects the cells against apoptosis [25]. In the first step of the study, it was determined that YAP is specifically phosphorylated by JNK1/2 in five previously unreported sites, T119, S138, T154, S317 and T362, in which each serine/threonine was followed by proline, enabling JNK-dependent phosphorylation [25]. Considering that UV is a potent activator of the JNK pathway, irradiation of HaCaT keratinocytes with 50 J/m^2^ UVC resulted in YAP phosphorylation in S317 and T362 residues by JNK1/2. To determine the role of JNK-phosphorylated YAP in UV-induced apoptosis, a comparison of cell death rate between control cells (without YAP silencing) and YAP-deficient cells was made, revealing that YAP silencing resulted in a higher cell death percentage than in control cells (70% vs. 40%, respectively), implicating that YAP phosphorylation in S317/T362 protects keratinocytes against UVC-induced apoptosis [25]. Despite these findings, other types of UVR have not been investigated in regard to the Hippo pathway, which may be surprising in terms of environmental UV hazards, compelling us to use UVB. In our study, exposure to UVB in human keratinocytes resulted in a nonsignificant stable increase in YAP mRNA in comparison to non-irradiated keratinocytes regardless of UVB dose. We also observed a dose-dependent increase in LATS1 and LATS2, which was not present after YAP silencing (Figure 3A,B). We hypothesize that exposure to UVB activates both kinases to phosphorylate YAP, thus preventing it from relocation into the nucleus and thereby inhibiting its binding with TEAD transcription factors, while in the absence of YAP LATS1/2, the function is abolished, but it is yet to be confirmed. Interestingly, no significant change in JNK1/2 mRNA was observed; however, there was a tendency towards a dose-dependent increase in keratinocytes without YAP silencing, while silencing of YAP diminished this effect, indicating reciprocal crosstalk between YAP and JNK1/2.

MAP4K4 is a Ste20p serine/threonine kinase, in which the function related to skin homeostasis is still evaluated; however, some recent insights provided evidence that MAP4K4 should be considered a component of the Hippo pathway. Meng et al. [26] showed that MAP4K kinases, including MAP4K4, phosphorylate LATS1/2 independently of MST1/2, thus displaying functional redundancy with MST1/2 [26]. We noticed that keratinocyte irradiation with UVB results in a dose-dependent increase in MAP4K4, but after YAP silencing, this effect was almost negligible (Figure 3E), suggesting that in response to cellular damage, YAP may be negatively controlled by MAP4K4, either via phosphorylation of LATS1/2 or direct phosphorylation, which needs to be determined in the future. Since genotoxic stress activates MAP4K4 by binding with p53 [41], it is possible that the observed dose-dependent upregulation of MAP4K4 is another compensatory mechanism inhibiting vicious YAP activity. Used doses of UVB radiation also significantly upregulated ABL1 mRNA expression levels in a dose-dependent manner, regardless of YAP silencing. This effect, however, was lessened in YAP-silenced keratinocytes (Figure 3F), implying a regulatory loop between YAP and ABL1. ABL1, being activated after DNA injury, phosphorylates YAP, which binds to suppressor protein p73, starting proapoptotic gene expression. Interestingly, Cottini et al. [42] showed that low YAP levels in multiple myeloma cell lines rescue cells from imatinib-induced ABL1-mediated apoptosis, demonstrating that YAP is necessary as a proapoptotic regulator for ABL1 [42]. While these results could not be extrapolated, they may be an explanation for lowered ABL1 mRNA levels after YAP silencing.

In the last step of our experiment, we decided to study the influence of narrowband UVB (311 nm) on Hippo signaling pathway components under the same conditions. Due to the fact that narrowband UVB is widely used in the therapy of psoriasis vulgaris with outstanding effects, along with recent reports about the involvement of YAP in psoriasis [43], we hypothesized that the antiproliferative effect achieved with narrowband UVB therapy could be YAP-driven. Alas, irradiation with increasing doses of narrowband UVB did not significantly change YAP mRNA expression in keratinocytes (Figure 2B). Additionally, irradiation with narrowband UVB resulted in increased expression of LATS1/2 in a dose-dependent manner in non-YAP-silenced keratinocytes (Figure 4A,B), similar to UVB irradiation (Figure 3A,B). Moreover, ABL1 mRNA expression after narrowband UVB was also increased, thus implying that the therapeutic effects of narrowband UVB rely, as suggested by Aufiero et al. [44], on apoptosis induction, but more detailed studies are needed to evaluate this relationship.

In addition to ongoing inquiries about the intricate mechanisms that regulate the Hippo pathway, it should be emphasized that targeting the Hippo pathway seems to be a promising method in the treatment of cutaneous tumors, particularly cutaneous melanoma [45]. As highlighted in recent studies, YAP activation promotes cell survival by upregulating anti-apoptotic factors, such as BCL-xL, and by inhibiting immune surveillance via PD-L1 expression [45,46,47]. The direct crosstalk between the Hippo and MAPK signaling pathways further complicates resistance, as it has been shown that actin cytoskeleton remodeling can promote YAP/TAZ nuclear accumulation and resistance to BRAF inhibitors [48]. Notably, targeting YAP-driven mechanisms, such as inhibiting actin polymerization or suppressing the gene *SLC35B2*, has shown promise in preclinical models, providing new potential strategies for overcoming therapy resistance [45,49].

To our best knowledge, no previous study assessed the influence of different doses of UVB in the context of Hippo pathway components, but the presented results need to be replicated in more detailed investigations using different cell lines and in vivo studies on human volunteers. Despite the presented findings, our study has multiple limitations. First of all, the expression of the Hippo pathway in cultured cell lines taken from human cutaneous squamous cell carcinoma should be studied instead of using commercially available cell lines. While there are several reports in which YAP expression was analyzed in SCC samples, other components and possible regulators of the Hippo pathway have not been investigated thoroughly. Secondly, our study was focused on the crude influence of UVB on the Hippo pathway, yet more research is needed to establish its expression under the influence of higher doses of UVB; we hypothesize that the used doses are not sufficient in terms of UV-induced carcinogenesis. Thirdly, the absence of cell survival and proliferation assays following UV treatment also limits the conclusions of the study. While our PCR analysis provides insights into the transcriptional regulation of YAP signaling, it does not directly assess the functional consequences of these changes on cell viability. Additionally, one of the most significant drawbacks of our study is the lack of methods assessing both YAP phosphorylation and its interaction with regulatory kinases. Finally, the influence of chronic exposure to UVR on Hippo pathway proteins needs to be assessed in further studies.

## 5. Conclusions

Overall, YAP mRNA expression does not significantly increase after exposure to UVB; however, upregulated expression of its proven (LATS1/2, JNK1/2) and presumed (MAP4K4, ABL1) regulators suggest that in a real-life setting, UV-induced dysregulation of the Hippo pathway may not be limited to YAP. Additionally, it is important to emphasize that exposure to UVB does not solely cause dysregulation of the Hippo pathway on its own but rather through crosstalk with other signaling pathways, particularly the p38 MAPK pathway, which plays a significant role in UVB-induced squamous carcinogenesis [50,51]. While the p38 MAPK pathway promotes cell survival by inducing cell cycle arrest, its activation may facilitate the development of cutaneous squamous cell carcinoma (cSCC) by promoting the expression of Hippo pathway target genes [52]. However, under certain conditions, p38 MAPK also induces the cytoplasmic translocation of TEAD, thereby inhibiting the nuclear accumulation of YAP [53]. It is necessary for more detailed studies to determine whether changes in Hippo pathway gene expression represent a compensatory mechanism in response to subsequent DNA damage or a pivotal step marking the transition from healthy skin to developing cancer.

## Figures and Tables

**Table 1:** TaqMan^®^ probes used for PCR.

Gene	TaqMan^®^ Probe
*GAPDH*	Hs02786624_g1
*YAP*	Hs00902712_g1
*LATS1*	Hs01125524_m1
*LATS2*	Hs01059009_m1
*JNK1*	Hs00255559_m1
*JNK2*	Hs01558224_m1
*ABL1*	Hs01104728_m1
*MAP4K4*	Hs01101394_m1

**Table 2:** Fold change of YAP and its regulators in A431 cell line. Data presented as fold change with geometric standard deviations. Data presented as fold change (geometric mean) with geometric standard deviations.

cSCC (A431)	YAP	LATS1	LATS2	JNK1	JNK2	ABL1	MAP4K4
Fold change	1.21	5.16	1.84	6.83)	3.62	2.93	1.38
Geometric SD	3.02	1.36	1.34	1.40	1.54	1.69	1.38
*p*-value	0.131	0.001	0.004	0.001	0.001	0.002	0.024

**Table 3:** Detailed mRNA expression of studied Hippo pathway regulators in primary epidermal keratinocytes under the influence of UVB. Data presented as fold change (geometric mean) with geometric standard deviations.

UVB Dose (J/cm^2^)/mRNA		No YAP Silencing					YAP Silencing				
		0.005	0.01	0.02	0.04	*p*-Value	0.005	0.01	0.02	0.04	*p*-Value
YAP	Mean	2.30	2.49	2.66	2.56	0.083	-				
	SD	1.34	1.22	2.18	1.50						
LATS1	Mean	2.04	2.50	4.57	5.67	0.033	1.47	2.02	1.83	2.68	0.329
	SD	1.44	1.11	2.25	2.62		1.69	1.43	1.94	2.22	
LATS2	Mean	3.03	4.17	10.92	14.97	0.016	1.64	3.31	3.36	4.91	0.042
	SD	1.27	1.63.2	2.52	4.24		1.78	1.32	2.11	2.26	
JNK1	Mean	2.95	3.90	5.50	4.04	0.223	1.63	1.51	1.39	1.82	0.391
	SD	1.39	1.46	2.58	5.06		1.57	1.33	1.64	1.48	
JNK2	Mean	1.52	2.15	2.77	3.00	0.209	0.91	1.40	0.96	1.52	0.853
	SD	1.49	1.57	2.38	2.15		2.02	2.41	1.73	1,94	
ABL1	Mean	3.60	4.45	10.30	10.66	0.039	3.82	5.15	5.41	7.88	0.003
	SD	1.33	1.47	2.71	4.67		1.28	1.54	1.63	1.83	
MAP4K4	Mean	1.90	2.70	6.73	8.62	0.027	1.41	1.46	2.03	3.07	0.253
	SD	1.61	1.58	2.35	3.42		1.72	2.01	1.94	1.98	

**Table 4:** Detailed mRNA expression of studied Hippo pathway regulators in primary epidermal keratinocytes under the influence of narrowband UVB. Data presented as fold change (geometric mean) with geometric standard deviations.

Narrowband UVB Dose (J/cm^2^)/mRNA		No YAP Silencing					YAP Silencing				
		0.01	0.02	0.05	0.1	*p*-Value	0.01	0.02	0.05	0.1	*p*-Value
YAP	Mean	1.36	1.21	1.33	1.56	0.164	-				
	SD	1.16	1.14	1.06	1.16						
LATS1	Mean	1.26	1.24	1.74	2.43	0.031	1.61	1.13	1.65	1.87	0.775
	SD	1.04	1.20	1.22	1.35		1.85	1.09	1.08	2.61	
LATS2	Mean	2.45	2.34	3.33	5.63	0.347	1.30	1,08	2.51	1.82	0.371
	SD	1.49	2.72	2.44	2.49		1.00	1.10	1.06	1.18	
JNK1	Mean	1.64	2.17	1.59	1.66	0.092	1.39	0.87	1.65	0.95	0.282
	SD	1.30	1.32	1.16	1.22		1.74	1.11	1.26	1.36	
JNK2	Mean	2.45	2.34	3.33	5.63	0.347	1.13	0.54	0.63	1.28	0.789
	SD	1.49	2.72	2.44	2.49		1.02	1.15	2.49	1.58	
ABL1	Mean	2.81	3.15	3.71	5.12	0.006	4.15	2.75	5.12	5.40	0.022
	SD	1.11	1.29	1.13	1.47		1.44	1.19	1.29	2.82	
MAP4K4	Mean	1.17	1.40	1.26	3.27	0.033	1.08	0.69	1.58	1.08	0.699
	SD	1.03	1.13	1.43	1.54		1.71	2.22	1.32	3.17	

## Data Availability

The data presented in this study are available on request from the corresponding author.

## Abbreviations

ABL1	V-abl Abelson murine leukemia viral oncogene homolog 1
JNK1/2	Jun N-terminal kinases
LATS1/2	Large tumor suppressor 1/2
MAP4K	Map kinase kinase kinase kinase
MAPK	Mitogen-activated protein kinase
MOB1	Monopolar spindle one binder kinase activator 1
MST1/2	Mammalian Ste20-like kinase 1/2
NMSC	Non-melanoma skin cancer
SCC	Squamous cell carcinoma
TAZ	Transcriptional coactivator with PDZ binding motif
TEAD	Transcriptional-enhanced associate domain
UVR	Ultraviolet radiation
YAP	Yes-associated protein
